# Identification of cuprotosis-mediated subtypes, the development of a prognosis model, and influence immune microenvironment in hepatocellular carcinoma

**DOI:** 10.3389/fonc.2022.941211

**Published:** 2022-08-30

**Authors:** Jingjing Xiao, Zhenhua Liu, Jinlong Wang, Shuaimin Zhang, Yi Zhang

**Affiliations:** ^1^ Department of Hepatobiliary Surgery, Guizhou Provincial People’s Hospital, Guiyang, China; ^2^ School of Clinical Medicine, Guizhou Medical University, Guiyang, China; ^3^ Department of Critical Care Medicine, Guizhou Provincial People’s Hospital, Guiyang, China

**Keywords:** hepatocellular carcinoma, cuprotosis, prognosis model, influence immune microenvironment, cuprotosis-mediated patterns-related genes

## Abstract

**Purpose:**

Cuprotosis is a newly discovered form of non-apoptotic regulated cell death and is characterized by copper-dependent and associated with mitochondrial respiration. However, the prognostic significance and function of cuprotosis-related genes (CRGs) in hepatocellular carcinoma (HCC) are unknown. This study aims to develop cuprotosis-mediated patterns-related gene (CMPRG) prediction models for the prognosis of patients with HCC, exploring the functional underlying the CRGs on the influence of tumor microenvironment (TME) features.

**Experimental design:**

This study obtained transcriptome profiling and the corresponding clinical information from the TCGA and GEO databases. Besides, the Cox regression model with LASSO was implemented to build a multi-gene signature, which was then validated in an internal validation set and two external validation sets through Kaplan-Meier, DCA, and ROC analyses.

**Results:**

According to the LASSO analysis, we screened out a cuprotosis-mediated pattern 5-gene combination (including PBK; MMP1; GNAZ; GPC1 and AKR1D1). A nomogram was constructed for the presentation of the final model. The ROC curve assessed the model’s predictive ability, which resulted in an area under the curve (AUC) values ranging from 0.604 to 0.787 underwent internal and two external validation sets. Meanwhile, the risk score divided the patients into two groups of high and low risk, and the survival rate of high-risk patients was significantly lower than that of low-risk patients (P<0.01). The risk score could be an independent prognostic factor in the multifactorial Cox regression analysis (P<0.01). Functional analysis revealed that immune status, mutational loads, and drug sensitivity differed between the two risk groups.

**Conclusions:**

In summary, we identified three cuprotosis-mediated patterns in HCC. And CMPRGs are a promising candidate biomarker for HCC early detection, owing to their strong performance in predicting HCC prognosis and therapy. Quantifying cuprotosis-mediated patterns in individual samples may help improve the understanding of multiomic characteristics and guide the development of targeted therapy for HCC.

## Introduction

Hepatocellular carcinoma is characterized by high malignancy, rapid progression, and poor prognosis (1). Worldwide, HCC has the second-highest mortality rate among cancers, and its incidence is increasing year by year (2). Under various treatments such as surgery, radiofrequency ablation, radiation therapy, and chemotherapy, HCC cells can still proliferate, invade, and metastasize by evading death (3); therefore, it is significant to find molecular markers and new targets for treatment that can predict patient prognosis. Cuprotosis, a newly identified mode of programmed cell death by TODD R. GOLUB’s team in 2022 (4), has been shown to occur through direct binding of copper to lipid acylated components of the TCA cycle, which leads to aggregation of lipid acylated proteins and loss of iron-sulfur cluster proteins, resulting in proteotoxic stress and ultimately cell death. FDX1 was also an upstream regulator of proteolipid acylation, and FDX1 and proteolipid acylation are critical regulators of cuprotosis. Cuprotosis will gradually become a hot topic of research in oncology. Jianghong Rao et al. (5) invented copper-depleting nanoparticles (CDN) to deplete copper in tumor and showed that the positive surface charge of CDN facilitates accumulation in mitochondria and local depletion of copper, which in turn induces apoptosis in triple-negative breast cancer cells. Some studies have shown that pancreatic cancer patients are higher serum copper levels, and copper has been shown to have tumor-promoting activity in cells (6, 7). Copper-chelation therapy in lung adenocarcinoma can prevent autophagy signal transduction, inhibit tumor cell proliferation and improve survival rate (8, 9). Most studies have shown that inhibition of cancer cell proliferation requires copper-binding and cellular uptake of copper complexes (10–13). At present, there is no article fully reporting the relationship between HCC and cuprotosis-mediated patterns-related genes.

The tumor microenvironment (TME) plays a critical role in tumor genesis and development in current studies (14). As cancer research has intensified, the role and relationship between cancer cells and immune cells have gradually been emphasized and confirmed as a new feature of cancer (15). It is clear that, in addition to cancer cells, the tumor microenvironment contains a range of immune cells, stromal cells, endothelial cells, and cancer-associated fibroblasts (14). Cancer cells can also evade immune surveillance and destruction using a range of mechanisms (16). tumor-infiltrating immune cells (TIICs) within the TME can predict the prognosis of cancer patients (16). Currently, most studies assess only one or two cuprotosis-related genes (CRGs) and cell types, whereas antitumor effects are characterized by many genes interacting synergistically. Therefore, a comprehensive understanding of the characteristics of multiple CMPRG-mediated TME cell infiltration in hepatocellular carcinoma may provide ideas for potential mechanisms of HCC tumorigenesis and prediction of response to immunotherapy.

Previous studies have confirmed that copper plays an essential role in tumorigenesis and tumor immune microenvironment. However, some functions of cuprotosis-mediated patterns-related genes in HCC remain unclear. Therefore, this study identified prognostic subtypes associated with cuprotosis patterns in HCC by exploring the expression levels of cuprotosis-mediated patterns-related genes in HCC and discussing the effects of these genes on tumor-associated pathways and tumor immune infiltration. Ultimately, a model to predict the prognosis of HCC was constructed and validated by an internal validation set and two external validation sets.

## Materials and methods

### Data collection and processing

The mRNA expression data and related clinical data of HCC were derived from GSE76427 dataset in GEO (https://www.ncbi.nlm.nih.gov/geo/) database and TCGA (https://portal.gdc.cancer.gov/) database. All mRNA Expression data were normalized using quantile normalization. To get TPMs we first used Cufflinks to generate FPKM (fragments per kilobase of transcript per million) and transformed these to TPM values, and calibration for batch effects was done with the ComBat algorithm from the sva library. After excluding patients with missing covariate information or no follow-up data, 491 HCC patients were included in the analysis (**Supplementary Table 1**). Clinical variables included age, sex, stage, follow-up time and survival status.

### Consensus clustering analysis

To identify clusters of highly cuprotosis-related, a consensus clustering algorithm was applied to determine the number of clusters in the TCGA-LUAD and GSE76427 dataset, undertaken using the ConsensuClusterPlus R package with 1000 permutations (17, 18). Here, K-means clustering was applied to sort CRGclusters into 9 clusters.

### Gene set variation analysis (GSVA), and CIBERSORTx immuno-infiltration analysis

GSVA was performed with the R package “gsva” to evaluate pathway enrichment for different CRGclusters. Then, underlying mechanisms were investigated within the “Molecular Signatures Database” of c2.cp.kegg.v6.2.symbols through gene set enrichment analysis GSEA with a Java program. Differences between different CRGclusters were analyzed using the R language “limma” package, and a p-value less than 0.05 was statistically significant.

CIBERSORTx (https://cibersortx.stanford.edu/) was used to estimate gene expression profiles and the abundance of member cell types in mixed cell populations using gene expression data. First, the normalized mRNA dataset was uploaded to CIBERSORTx, and the number of permutation permutations was selected 1,000 times using the web analysis tool to obtain the content of 22 immune cell types in each sample. Then, the composition of various immune cells was compared between different CRGclusters.

### DEG identification and function notes

The R software limma package was used to standardize the data and screen the differential genes, and the | log FC | > 1 and corrected P < 0.05 were used as the thresholds to screen the differential genes. In order to further explore the potential biological function of DEG related to cuprotosis patters, GO, and KEGG analysis was performed on the screened differentially expressed hub genes using DAVID online tool. P < 0.05 indicated that the difference was statistically significant. Column and chord maps were plotted using the R language ggplot package

### Construction of the cuprotosis-mediated patterns-related prognostic CMPRG_score

The cuprotosis score was calculated to quantify the cuprotosis pattern of a single tumor. Firstly, the DEGs expression of cuprotosis pattern was analyzed using R language “survival” package by single factor COX analysis. According to P < 0.05, the genes related to the prognosis of cuprotosis patterns were screened. Next, the Cox regression model with LASSO was used to determine the critical genes for the prognosis of cuprotosis pattern according to the best Akaike Information Criterion (AIC) (19, 20). The prognostic risk model was constructed, and CMPRG_score was calculated as follows: CMPRG_score =Σ(Exp * coefi) n, where Coefi and Expi represent the regression coefficients and expression levels of each CMPRG, respectively. Secondly, according to the expression of CMPRGs in prognosis, the patients were divided into different subtypes (CMPRG subtype A, CMPRG subtype B, CMPRG subtype C) by unsupervised clustering analysis. Finally, all HCC patients were randomly divided into training sets and test sets according to 1: 1. With the median risk value as the limit, the patients were divided into high and low-risk groups in the total sample, training set and test set and subjected to Kaplan-Meier survival analysis. Concurrently, the “ggplot2” package in R language was used for principal component analysis (PCA), and the correlation between geneCluster, CRGCluster, CMPRG_score and survival status were visualized by “ggalluvial” package.

### Assessment of immune status and cancer stem cell (CSC) index between high- and low-risk groups

To assess the proportion of TIIC in TME, this study used the CIBERSORT deconvolution algorithm to estimate the percentage of different immune cells and the variability of immune cells between high and low-risk groups of liver cancer samples, and the ESTIMATE algorithm was used to calculate the immune and stromal scores for each patient (21, 22). Then, the correlation between CMPRG_score and 22 infiltrating immune cells was explored (p<0.05). In addition, we analyzed the relationship between the two risk groups and CSC.

### Mutation and drug sensitivity analysis

To identify somatic mutations in HCC patients between the high and low-risk groups, we annotated the mutation data in the TCGA database into MAF format using the R language “maftools” package and calculated the tumor mutational load (TMB) score for each HCC patient in the high and low-risk groups. To explore the differences in the therapeutic effects of chemotherapeutic agents in the two groups, we calculated the semi-inhibitory concentration (IC50) values of common chemotherapeutic agents for HCC using the “pRRophetic” package.

### Construction and validation of nomograms

We analyzed the obtained CMPRG_score combined with clinical information and performed univariate and multivariate COX analysis to determine the potential value of the CMPRG_score in clinical application. Then, the nomogram for predicting the HCC patients at 1, 3 and 5 years was constructed using the “rms” package according to the contribution of each variable (magnitude of regression coefficients). The accuracy of the nomogram was assessed by applying the ROC and clinical decision curve (DCA), and the calibration curve of the nomogram prediction model was also estimated for consistency.

### Statistical analysis

All statistical analyses were performed using R version 4.1.2. Statistical tests were all considered statistically significant at P<0.05, and estimates were considered significant at a CI of 95%. The flowsheet of this program is shown in **Figure S1**.

## Results

### Genetic and transcriptional alterations of CRGs in HCC

**Figure 1:**
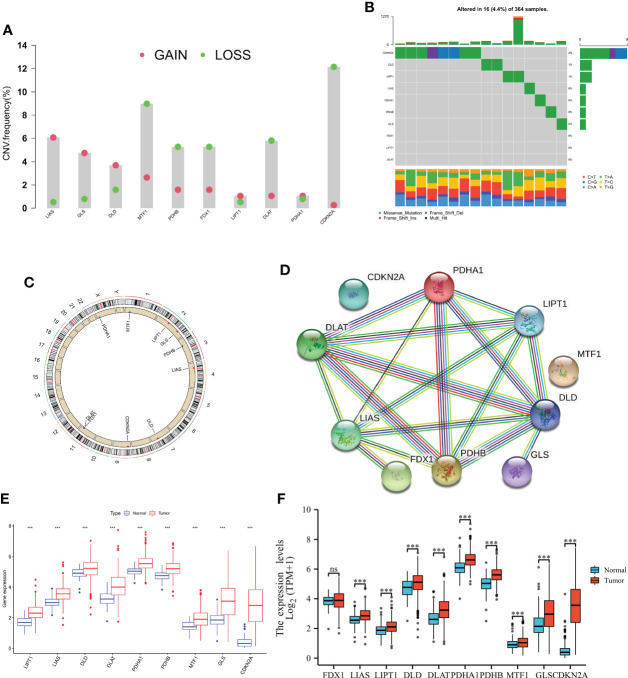
Genetic and transcriptional alterations of CRGs in HCC. **(A)** Frequencies of CNV gain, loss, and non-CNV among CRGs. **(B)** Mutation frequencies of 10 PRGs in 364 patients with HCC, respectively, from the TCGA cohort. **(C)** Locations of CNV alterations in PRGs on 23 chromosomes. **(D)** cuprotosis-related genes are mapped on proteins in the human protein-protein interaction network. **(E, F)** Expression distributions of 10 CRGs between normal and HCC tissues from the TCGA and UCSCXENA database, respectively. NS, Not statistically significant. *P < 0.05, **P < 0.01, ***P < 0.001.

To explored somatic copy-number alterations in these CRGs (**Supplementary Table 2**), among them LIPT1; LIAS; DLD; PDHA1 and GLS were found to have increased extensive copy number variation (CNV); while FDX1; DLAT; PDHB; MTF1 and CDKN2A showed decreased CNV (**Figure 1A**) and their CNV locations on chromatin were analyzed (**Figure 1C**). Next, we assessed the relationship between somatic mutation rate and CRGs in the HCC cohort of TCGA. While we do also observe that 16 of 365 samples (13.63%) were mutated in CRGs. Among them, CDKN2A had the highest mutation frequency, but no FDX1, LIPT, and DLAT mutations were found in any of the HCC samples (**Figure 1B**). Protein interaction network analysis of DEGs was performed using STRING online software (**Figure 1D**). At the expression level, nine CRGs were identified in the TCGA and UCSCXENA (https://xenabrowser.net/hub/) databases, respectively, that were able to distinguish between normal and tumor samples (**Figures 1E, F**), and were all upregulated in HCC samples compared to normal samples.

### Identification of cuprotosis subtypes in HCC

The gene expression matrices of both TCGA and GSE76427 datasets were combined into a single matrix for further analysis. We then carried out topological 10 CRGs interaction network analysis in the merge cohort (**Figure S1A**), and then Kaplan-Meier survival analyses showed that among the 10 CRGs, 9 CRGs were significantly correlated with overall survival in HCC patients except for FDX1, which was not correlated with overall survival (**Figure S1B–J**).

**Figure 2:**
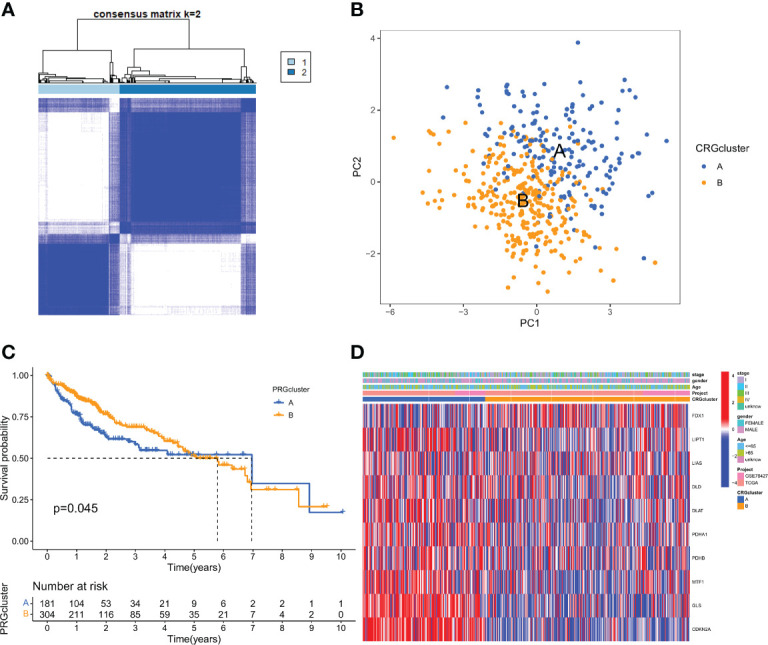
CRG subtypes and clinicopathological and biological characteristics of two distinct subtypes of samples divided by consistent clustering. **(A)** Consensus matrix heatmap defining two clusters (k = 2) and their correlation area. **(B)** PCA analysis showing a remarkable difference in transcriptomes between the two subtypes. **(C)** Univariate analysis showing 10 CRGs related to the OS time. **(D)** Differences in clinicopathologic features and expression levels of CRGs between the two distinct subtypes.

To understand the expression characteristics of CRGs in HCC, we performed unsupervised clustering analysis to categorize the patients with HCC based on the expression profiles of the 10 CRGs (**Figure S2**). Subsequently, cuprotosis mediation patterns were analysed by unsupervised clustering analysis based on these genes. According to the CDF curves of the consensus score, we selected the k = 2 as the optimal division for sorting the entire cohort into sub-types A (n = 182) and B (n = 304) (**Figure 2A**; **Supplementary Table 3**). Principal components analysis (PCA) highlighted significant differences between the two subtypes (**Figure 2B**). The Kaplan-Meier curves showed that CRGcluster B was associated with better survival than CRGcluster A (**Figure 2C**). Furthermore, A heat map was constructed to illustrate the associations between the CRGclusters, CRGs gene expression level, and clinicopathologic characteristics (**Figure 2D**).

### Characteristics of the TME in distinct subtypes

**Figure 3:**
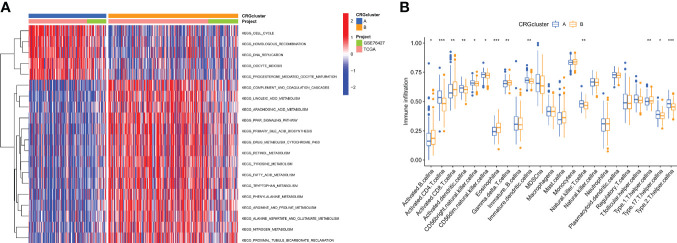
Correlations of tumor immune cell microenvironments and two HCC subtypes. **(A)** GSVA of biological pathways between two distinct subtypes, in which red and blue represent activated and blue inhibited pathways, respectively. **(B)** Abundance of 22 infiltrating immune cell types in the two HCC subtypes. *P < 0.05, **P < 0.01, ***P < 0.001.

GSEA enrichment analysis showed that subtype A was mainly enriched in the cell cycle, homologous recombination, DNA replication, oocyte meiosis, and progesterone-mediated oocyte maturation pathways. Subtype B was enriched primarily in the complement and coagulation cascades, linoleic acid metabolism, and arachidonic acid metabolism pathways (**Figure 3A**). It was, therefore, suggested that subtype A is associated with the activation of the cell proliferation process, and subtype B is associated with immune response. To further confirm the correlation between different subtypes and the immune microenvironment, the CIBERSORT algorithm was used to analyze the differences of 23 kinds of immune cells between HCC samples of different subtypes. We observed that most immune cells were significantly different between different subtypes. Among them are Activated CD4 T cell Activated dendritic cell, CD56bright natural killer cell, CD56dim natural killer cell, Immature dendritic cell, Type 17 T helper cell, Type 2 T helper cell in subtype A had significantly higher infiltration. In the B subtype, Activated B cells, Activated CD8 T cells, Type 1 T helper cells, and Gamma delta T cells had significantly higher infiltration (**Figure 3B**).

### Identification of gene subtypes based on cuprotosis-related DEGs

**Figure 4:**
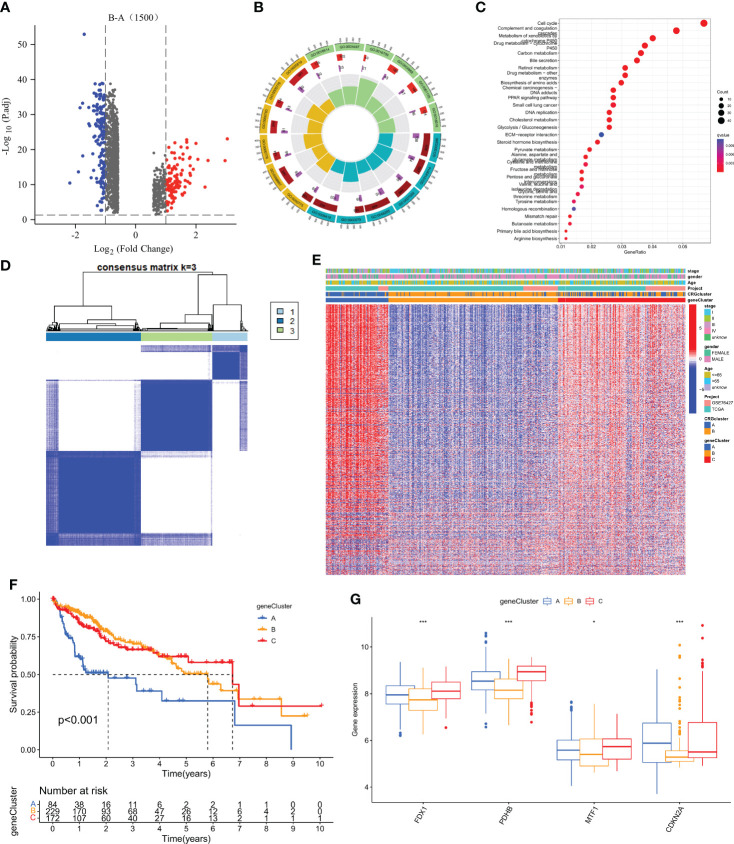
Identification of gene subtypes based on DEGs. **(A)** 1500 DEGs between different subtypes of volcano map. **(B, C)** GO and KEGG enrichment analyses of DEGs among two cuprotosis subtypes. **(D)** Consensus matrix heatmap defining two clusters (k = 3) and their correlation area. **(E)** Relationships between clinicopathologic features and the three gene subtypes. **(F)** Kaplan–Meier curves for OS of the three gene subtypes (log-rank tests, *p* < 0.001). **(G)** Differences in the expression of 4 CRGs among the three gene subtypes. *P < 0.05, **P < 0.01, ***P < 0.001.

To explore the potential biologic functions of cuprotosis patterns, we identified 1500 cuprotosis-related subtype differential genes (DEGs) using the R language “limma” package (**Table S4**). And the filter standard was set at |log2FC|≥1 and the adjusted p value (FDR) < 0.05. Together, there have 275 DEGs between the two subgroups (**Figure 4A**). Then, GO, and KEGG enrichment analyses were performed (**Figure 4B**; **Table S5**). KEGG pathway enrichment analysis on the DEGs showed that the Cell cycle, complement, and coagulation cascade pathway were enriched (**Figure 4C**; **Table S4**). Next, an unsupervised clustering analysis was performed based on the 1500 differential genes associated with cuprotosis-related subtypes. The unsupervised clustering algorithm also revealed three different cuprotosis-mediated patterns-related gene subtypes. We named cuprotosis-mediated patterns-related genes clusters A-C (**Figure 4D**), with three other gene clusters having different characteristic genes. Kaplan-Meier curves showed that the analysis of patients with HCC with gene subtype C had the worst prognosis and those with gene subtype B had a better prognosis (**Figure 4E**); moreover, the cuprotosis-mediated patterns-related genomic B subtypes correlated with advanced TNM stage, age and gender (**Figure 4F**). Among the three cuprotosis-mediated patterns-related gene clusters, significant differences were observed in the expression of four of the cuprotosis-mediated patterns-related genes (**Figure 4G**).

### Construction of a prognostic risk assessment model for hepatocellular carcinoma

**Figure 5:**
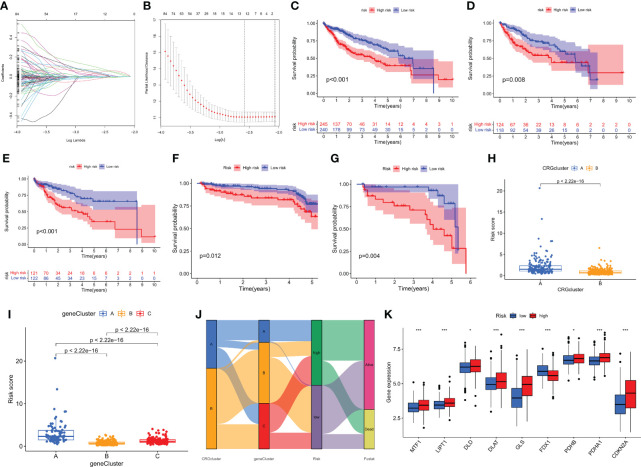
Construction of the CMPRG_score. **(A, B)** The LASSO regression analysis and partial likelihood deviance on the prognostic genes. **(C–G)** Kaplan–Meier analysis of the OS between the three groups in the all sample, training set, testing set, GSE14520, and GSE116174 cohorts, respectively. **(H)** Differences in CMPRG_score between pyroptosis subtypes. **(I)** Differences in CMPRG_score between gene subtypes. **(J)** Alluvial diagram of subtype distributions in groups with different CMPRG_score and survival outcomes. **(K)** Differential expression of CRGs at different high and low CMPRG_score.

To further distinguish the heterogeneity of cuprotosis-mediated patterns-related genes in HCC individuals, a scoring system was constructed to quantify the regulatory patterns of CMPRGs in individual patients with hepatocellular carcinoma, called CMPRG_score, based on the genes associated with phenotypic differences in three gene clusters. First, we randomly divided the patients into a training group (n = 243) and a test group (n = 242) using the “caret” package in the R language in a 1:1 ratio. Univariate Cox regression analysis and Kaplan-Meier survival analysis were performed on 942 differential genes in the three gene clusters, and the overfitted genes were further removed by LASSO regression, and finally, five genes (PBK; MMP1; GNAZ; GPC1; AKR1D1) were identified by multivariate Cox regression analysis for prognostic model construction (**Figures 5A, B**). The risk coefficients for the genes were 0.2391, 0.1636, 0.1604 0.2109, and -0.1474, respectively. The risk score prognostic model was constructed as follows: CMPRG_score = (0.2391* expression of PBK) + (0.1636*expression of MMP1) + (0.1604* expression of GNAZ) + (0.2109* expression of GPC1) + (-0.1474* expression of AKR1D1) (**Table S6**).

The Kaplan-Meier survival curves showed that the survival rate of low-risk patients was significantly higher than that of high-risk patients in the overall sample, training set, validation set, and two external validation cohorts (GSE14520 and GSE116174), respectively (p< 0.01; **Figures 5C–G**), based on the median risk score dividing all samples into high- and low-risk groups. Meanwhile, we observed a significant difference in CMPRG_score between ccuprotosis-related geneclusters, and genecluster B had the lowest CMPRG_score, and genecluster A subtype had the highest CMPRG_score (**Figure 5H**). Here, it was also found that the CMPRG_score was significantly different among the different CRGclusters, with the CRGcluster A subtype having a significantly higher CMPRG_score than CRGcluster B (**Figure 5I**). We showed the changes in CRGclusters, CRGscore, gene cluster, and survival status using Sankey plots (**Figure 5J**). 10 cuprotosis-related genes differed significantly in high and low-risk groups (**Figure 5K**).

To validate the predictive performance of CMPRG_score, the risk scores were also divided into high and low-risk groups according to the validation and test sets, and **Figures S2B, C, E, F, and H, I** show the dynamic distribution of CMPRG_score and OS survival status in the overall sample, training set, and test set, respectively, all of which yielded consistent results. It shows that CMPRG_score have good performance in predicting the prognosis of HCC patients. The heat map also showed the expression of the five genes in the model in the high and low-risk groups, which performed consistently in the overall sample, training set, and test set (**Figure S2A, D, G**).

### Evaluation of TME between the high- and low-risk groups

We performed the CIBERSORT algorithm to assess the relationship between CMPRG_score and immune cell abundance, and the results showed that CMPRG_score was significantly and positively correlated with M2 macrophages, neutrophils (**Figure S3A, B**). We also observed that a low CMPRG_score was strongly correlated with a low stromal score (**Figure S3C**). Also, we evaluated the relationship between four genes in the prognostic model and immune cells, and we observed that most immune cells were less correlated with these four genes (**Figure S3D**).

### Relationship between CRG_score and CSC index, mutation and drug sensitivity analysis

**Figure 6:**
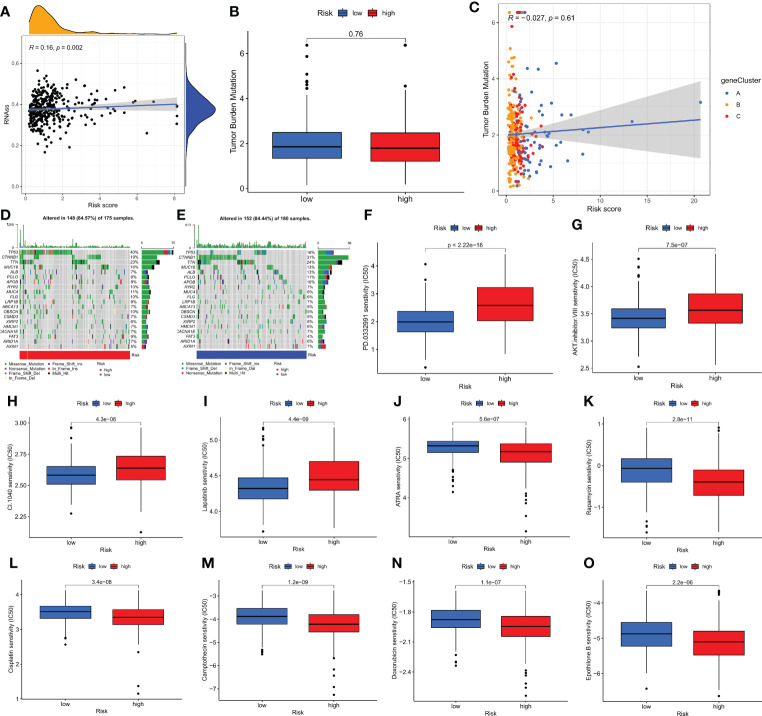
Comprehensive analysis of the CMPRG_score in HCC. **(A)** Relationships between CMPRG_score and CSC index. **(B)** TMB in different CMPRG_score groups. **(C)** Spearman correlation analysis of the CMPRG_score and TMB. **(D, E)** The waterfall plot of somatic mutation features established with high and low CMPRG_score. **(H–O)** Relationships between CMPRG_score and chemotherapeutic sensitivity.

In our study, we performed Pearson’s correlation analysis to estimate the correlation of CMPRG_score and CSC index values for the relapse, metastasis and chemoresistance of HCC. **Figure 6A** shows the results of linear correlation between CMPRG_score and CSC index, and we conclude that CMPRG_score was positively correlated with CSC index (R = 0.16, p=0.002), indicating that higher CMPRG_score was associated with more pronounced stem cell characteristics and a higher degree of cell differentiation in their HCC cells.

Increasing evidence suggests that Tumor mutational burden (TMB) can be used as a predictive marker for immunotherapy, and higher TMB may be associated with more significant immunotherapy benefits. Our analysis of mutation data from the TCGA cohort showed no difference in TMB scores between high and low-risk groups (**Figure 6B**), implying that there may be small immunotherapy responsiveness in high and low-risk populations. Spearman correlation analysis showed no correlation between TMB and risk scores in the geneCluster group (p=0.16) (**Figure 6C**). We then analyzed the distribution of somatic mutations between the high and low CMPRG_score groups in the TCGA-LIHC cohort. The top ten mutated genes in the high- and low-risk groups were TP53, CTNNB1, TTN, MUC16, ALB, PCLO, APOB, RYR2, MUC4, and FLG, respectively (**Figures 6D, E**). Patients in the high-risk group had a higher rate of TP53 mutations than those in the low-risk group, while CTNNB1 had a higher rate of mutations in patients in the low-risk group. Next, we selected chemotherapeutic agents currently used to treat HCC patients to assess the difference in sensitivity to these agents between the low- and high-risk groups.

Interestingly, we found lower IC50 values for ATRA, Rapamycin, Camptothecin, doxorubicin, cisplatin and Epothilone. B in patients in the high-risk group; while in patients in the low-risk group PD.0332991, AKT.inhibitor.VIII, CI.1040, and Lapatinib chemotherapeutic agents had lower IC50 values. In conclusion, these results suggest that CMPRG is associated with drug sensitivity (**Figures 6F–O**).

### Clinical correlation analysis of prognostic CMPRG_score, construction and validation of nomogram

**Figure 7:**
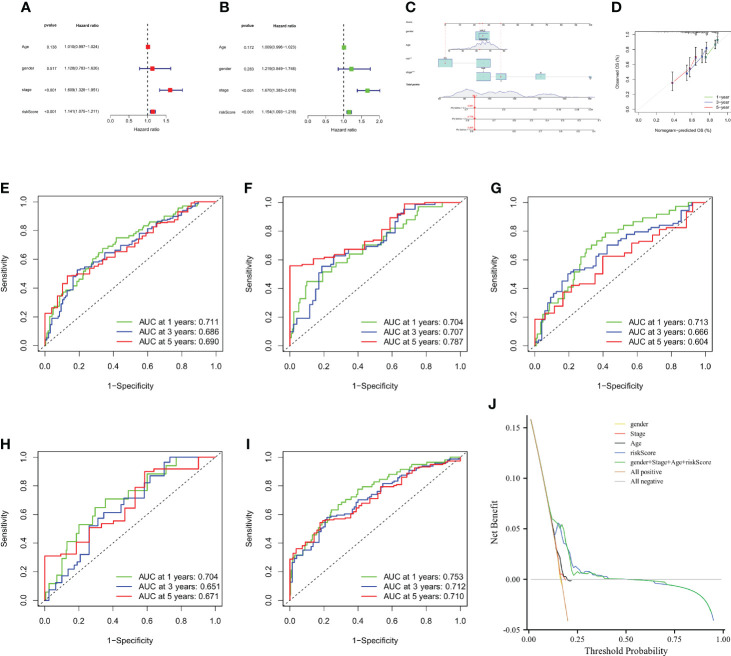
Construction and validation of a nomogram. **(A, B)** CMPRG_score and clinical characteristics of HCC were analyzed by univariate analysis and multivariate analysis. **(C)** Nomogram for predicting the 1-, 3-, and 5-year OS of HCC patients in the all sample. **(D)** Calibration curves of the nomogram for predicting of 1-, 3-, and 5-year OS in the TCGA and GSE76427 cohorts. **(E–G)** ROC curves for predicting the 1-, 3-, and 5-year ROC curves in the all sample, training, and testing sets. **(H, I)** ROC curves for predicting the 1-, 3-, and 5-year ROC curves in the GSE14520 (n = 221) and GSE116174 (n=64) cohorts. **(J)** Decision-making curve.

To investigate the effect of CMPRG_score on clinical characteristics, univariate Cox analysis showed that Stage staging and riskScore were associated with OS in HCC patients (**Figure 7A**). Further multifactorial Cox regression analysis showed that riskScore was an independent risk factor affecting OS in HCC patients (HR = 1.154, 95% CI: 1.093 to 1.218, P<0.001) (**Figure 7B**).

Considering the inconvenient clinical application of CMPRG_score in predicting the prognosis of HCC patients, we further constructed a nomogram of 1-, 3-, and 5-year overall survival of HCC patients based on the four clinical factors of CMPRG_score/stage/Age/gender (**Figure 7A**). Calibration plots of the predicted versus actual values of the 4-factor line plot model showed high agreement between the 1-, 3-, and 5-year OS of the line plot prognostic model and the accurate results (**Figure 7B**). In addition, our AUC curve results on the nomogram model showed that the 1-, 3-, and 5-year AUC values were 0.711, 0.686, and 0.690 in the overall sample (**Figure 7C**), and 0.704, 0.707, and 0.787 in training set for the 1-, 3-, and 5-year AUC values (**Figure 7D**), and the 1-, 3-, and 5-year AUC values in the test set were 0.713, 0.666, and 0.604 in the test set (**Figure 7E**), and the 1-, 3-, and 5-year AUC values were 0.704, 0.651, and 0.671 in GSE14520 cohorts (**Figure 7F**); and the 1-, 3-, and 5-year AUC values were 0.753, 0.712, and 0.710 in GSE116174 cohorts (**Figure 7G**). In conclusion, the 1-, 3-, and 5-year AUCs of the prognostic risk model in the overall sample, the training set, test set, and the two external validation cohorts (GSE14520 and GSE116174) achieved high accuracy, and the model was highly reliable. After assessing the model’s predictive accuracy, we further evaluated whether the addition of the four clinical factors could benefit HCC patients in clinical practice. The decision curve analysis (DCA) of the CMPRG_score assessed the net benefit to patients, and the more excellent the net benefit, the better the predictive performance of the prognostic risk model (**Figure 7H**).

## Discussion

Studies have concluded that cell death is mainly classified as apoptosis and necrosis (23). As research continues, cells can also die through autophagy, distension, scorch death, and iron death (24, 25). In addition, cuprotosis is one of the copper-dependent forms of programmed cell death discovered in 2022 (4), which is involved in various diseases and is expected to be a new modality for tumor treatment. It was found that a complex network of genes regulates the process of cellular cuprotosis. Here, it was shown that cuprotosis and immune cells are closely linked (26–28). Nevertheless, the role of CMPRGs in HCC has not been systematically elucidated. In this study, we used public databases to reveal the heterogeneity of HCC through consistent clustering analysis and construct prognostic models of genes associated with cuprotosis patterns and explore the relationship between iron death and clinical features and immune microenvironment.

Numerous studies have revealed the indispensable role of copper-mediated cell death in innate immunity and antitumor effects (29–31). However, most studies have focused on TME cells from a single CRG. Therefore, the overall disease effects and TME infiltration characteristics mediated by the combined effects of multiple CRGs have not been fully elucidated. Our study results reveal overall changes in CMPRGs at the transcriptional and genetic levels in HCC, identifying two distinct molecular subtypes based on 10 CRGs, with patients in subtype A having a better prognosis and tumor staging than those in subtype B. There are also significant differences in TME characteristics between the two, with subtype B also characterized by significant immune activation. In addition, mRNA transcriptome differences between cuprotosis subtypes were significantly associated with CRG and immune-related biological pathways. Here, we identified three genetic subtypes based on the DEG between the two cuprotosis- related subtypes. Our findings suggest that CMPRG may serve as a predictor for assessing clinical outcomes in HCC. Interestingly, we constructed good predictive prognostic models using CMPRG_score and validated the predictive power of the models by ROC, DCA and consistency calibration curves. Cuprotosis-mediated patterns characterized by immune activation and suppression showed lower and higher CMPRG_score, respectively. HCC patients with high and low CMPRG_score showed significant differences in clinicopathological characteristics, prognosis, mutations, TME, CSC index and drug sensitivity. Finally, by integrating gender, age, CMPRG_score and tumor stage, we created a nomogram that further improves the model’s performance and can guide the use of CMPRG_score in the clinic.

This study collected ten genes associated with cuprotosis by literature mining and analyzed them as such targets. Among the five differentially expressed prognostic genes associated with cuprotosis-mediated patterns, PBK, MMP1, GNAZ, and GPC1 were risk factors, and AKR1D1 was protective. PBK is a class of DNA damage repair factors and a member of the MAPPK family (32). PBK expression was found to be upregulated in various malignancies to varying degrees (33, 34). The lung cancer study confirmed that PBK was highly expressed in lung cancer tissues, and the prognosis of 119 lung cancer patients was correlated with PBK by statistical analysis. Patients with high PBK expression had poor prognosis and high tumor recurrence rate, suggesting that PBK is a predictor of lung cancer prognosis (35). MMP⁃9 is a gelatin-like matrix metalloproteinase with 13 exons and nine introns involved in the degradation of the tumor basement membrane and, thus, tumor metastasis (36, 37). In several studies of cervical cancer, MMP⁃9 has been shown to play an essential role in the invasion and metastasis of cervical cancer (38–40). Recombinant Glypican 1 (GPC1) is a member of the phosphatidylinositol glycan family of acetyl heparan sulfate glycoproteins (41). GPC1 expression level in serum of pancreatic ductal adenocarcinoma was 100% specific and sensitive for the diagnosis of early pancreatic cancer, and it is an ideal marker for early pancreatic cancer (42). In a study by Sidong Wei et al., serum GPC1 expression levels were a prognostic marker for hepatocellular carcinoma (43). G protein subunit alpha Z (GNAZ) is a potential oncogene in hepatocellular carcinoma, promoting tumor proliferation through cell cycle arrest, apoptosis, migration and invasion (44). Steroid 5β-reductase (AKR1D1) is an important molecule involved in endogenous glucocorticoid inactivation and catalyzes bile acid synthesis (45). Jeremy W Tomlinson et al. found that AKR1D1 expression was downregulated with the progression of adipose lesions in NAFLD (46). The above results suggest that PBK, MMP1, GNAZ, GPC1 and AKR1D1 can influence cancer development by regulating the cuprotosis process. However, their role in hepatocellular carcinoma is unclear. In our study, we found that they are closely related to the prognosis of HCC and may be important regulators of HCC development.

Failure to respond to conventional chemotherapy agents is a major barrier to the successful treatment of HCC (47). Despite recent advances in immunotherapy, the prognosis of HCC patients remains heterogeneous, highlighting the critical role of TME in HCC tumorigenesis and progression (48). We found that CMPRG_score was significantly correlated with M2 macrophages and neutrophils by correlation analysis of CRG_score with immune cells. M2 macrophages express anti-inflammatory cytokines, scavenging receptors, and angiogenesis in the tumor microenvironment, which can reshape the tumor microenvironment to suppress the immune response and thus promote tumor progression (49). Several previous studies have shown that M2-type macrophages promote the migration and invasion of tumor cells and enhance EMT (50–52). Infiltration of M2 macrophages in TME increases the metastasis of HCC, which is associated with poor prognosis in patients with HCC (53). Recent studies have shown that neutrophils are an essential component of infiltrating immune cells in TME, involved in tumor progression and inflammatory response, and associated with tumor resistance to chemotherapy and checkpoint blockade (54, 55). Zhou et al. (56) showed that neutrophils secrete CC chemokine ligand 2 (CCL2) and CC chemokine ligand 17 (CCL17), which mediate the infiltration of M2 macrophages and regulatory T cells into tumors and promote the development of HCC. A transparent zebrafish larval glioblastoma study demonstrates that neutrophils promote cancer cell proliferation in early TME in the brain (57). These findings are consistent with our findings that patients with high CMPRG_score have worse prognosis and immunotherapy responsiveness, and it can be speculated that M2 macrophages and neutrophils may also be potential immunotherapy targets for patients with high-risk CMPRG_score. Studies have confirmed that sorafenib is currently the first-line treatment for advanced HCC, while the combination of doxorubicin and cisplatin have prolonged survival in HCC treatment (58). However, targeted therapy for HCC is highly resistant and has a high individual non-response rate (59). Our study also confirmed no difference between high and low-risk groups for sorafenib, which is consistent with previous studies.

Undeniably, there are some limitations in the prognostic model of HCC based on public databases. Firstly, the current HCC database is not complete in terms of clinical characteristics, including pathological classification, surgery, radiotherapy, HBV status, smoking, and alcohol consumption, so these characteristics were not included in the independent predictive analysis of this study, which may limit the application of this prognostic model. Secondly, as this study is based on data mining analysis, some of the findings still need further validation in future functional experiments.

## Conclusions

In our comprehensive analysis, we identified three cuprotosis-mediated patterns in HCC, and cuprotosis-mediated patterns-related genes are revealed a broad range of regulatory mechanisms that influence the tumor immune microenvironment, clinicopathological features, and prognosis. The predictive prognostic model constructed based on CMPRG_score has high sensitivity and specificity and may provide a new entry point for immune and targeted therapy and prognostic assessment for patients with HCC.

## Data availability statement

The datasets presented in this study can be found in online repositories. The names of the repository/repositories and accession number(s) can be found in the article/**Supplementary Material**.

## Author contributions

JX, JW, and ZL: Conception/design, Provision of study material, Data analysis and interpretation, and Manuscript writing. JX, JW, ZL, and YZ: Conception/design, Provision of study material, and Data analysis and interpretation. JX, JW, SZ, and YZ: Conception/design. All authors read and approved the final version of the manuscript.

## Acknowledgments

This study was supported by the Youth Foundation of Guizhou Provincial People’s Hospital (GZSYQN [2018]01); We thank the TCGA team of the National Cancer Institute for using their data.

## Conflict of interest

The authors declare that the research was conducted in the absence of any commercial or financial relationships that could be construed as a potential conflict of interest.

## Publisher’s note

All claims expressed in this article are solely those of the authors and do not necessarily represent those of their affiliated organizations, or those of the publisher, the editors and the reviewers. Any product that may be evaluated in this article, or claim that may be made by its manufacturer, is not guaranteed or endorsed by the publisher.

## Abbreviations

CRGs, cuprotosis-related genes; CMPRG, cuprotosis-mediated patterns-related gene; TME, tumor microenvironment; HCC, Hepatocellular carcinoma; CSC, cancer stem cell; TIICs, tumor-infiltrating immune cells; TCGA, The Cancer Genome Atlas; GEO, gene expression omnibus; CDF, cumulative distribution function; DEGs, Differentially Expressed Genes; ROC, receiver operating characteristic; FPKM, Fragments Per Kilobase Million; GSVA, Gene set variation analysis; TMB, tumor mutation burden; CNV, copy number variation; ssGSEA, single-sample gene set enrichment analysis; PCA, principal component analysis; MAF, mutation annotation format; AIC, Akaike information criterion; CIBERSORT, Cell type identification by estimating relative subsets of RNA transcripts.
