# Supplementary material for: Identification of cuprotosis-mediated subtypes, the development of a prognosis model, and influence immune microenvironment in hepatocellular carcinoma
Source: Front Oncol. 2022 Aug 30;12:941211. doi: 10.3389/fonc.2022.941211 (PMC9468823; doi:10.3389/fonc.2022.941211)

**Supplementary Figure**

**FIGURE S1** | The entire analytical process of the study.

**FIGURE S2** | Interactions among CRGs in HCC **(A)**. The line connecting the PRGs represents their interaction, with the line thickness indicating the strength of the association between CRGs. Green and pink represent negative and pink positive correlations, respectively. . Kaplan-Meier analysis showed that high expression of CDKN2A **(B)**, DLAT **(C)**, DLD **(D)**, GLS **(E)**, LIPT1 **(G)**, PDHA1 **(H)**, MTF1 **(I)** and PDHB **(J)** was correlated with poor OS, while high expression of LIAS **(F)** was associated with poor OS

**FIGURE S3** | Unsupervised clustering of c**uprotosis**-related genes and Consensus matrix heatmaps for k=3-9.

**FIGURE S4** | Evaluation of the TME between the two groups. **(A-B)** Correlations between CMRPG_score and immune cell types. **(C)** Correlations between CRG_score and both immune and stromal scores. **(D)** Correlations between the abundance of immune cells and five genes in the proposed model.

**FIGURE S5** | **(A-C)** Ranked dot and scatter plots showing the CMRPG_score distribution and patient survival status in the overall sample. **(D-F)** Ranked dot and scatter plots showing the CMRPG_score distribution and patient survival status in the training set. **(G-I)** Ranked dot and scatter plots showing the CMRPG_score distribution and patient survival status in the testing set.

**Figure S1**

**Validation of CMRPG_score**

**HCC samples from two datasets**

**dentification of cuprotosis subtypes**

**Correlation of CMRPG_score with clinical characteristics and survival**

**Subtype A Subtype B**

**Unsupervised clustering for cuprotosis subtypes-related genes**

**Identification of c subtypes-related genes**

**Characteristics of TME in distinct subtypes**

**Correlation of CMRPG_score with TME**

**Correlation of CMRPG_score with HCC index**

**Correlation of CMRPG_score with mutation and drug susceptibility analysis**

**Gene subtype A**

**Gene subtype B**

**Development of a prognostic nomogram**

**LASSO and multivariate Cox regression analyses for quantifying cuprotosis pattern**

**Gene subtype C**


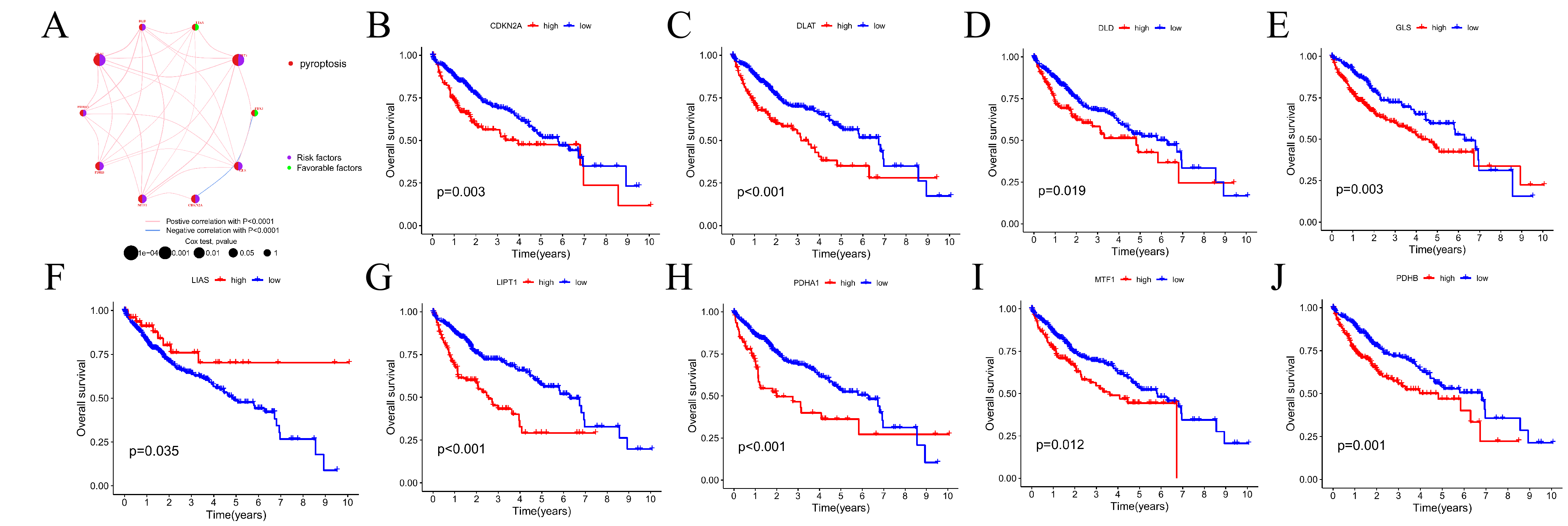


**Figure S2**

**Figure S3**


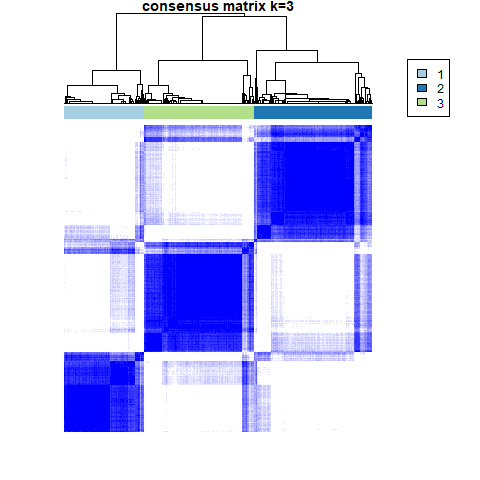

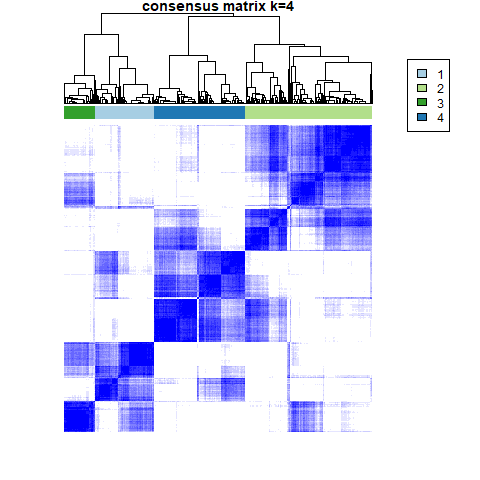

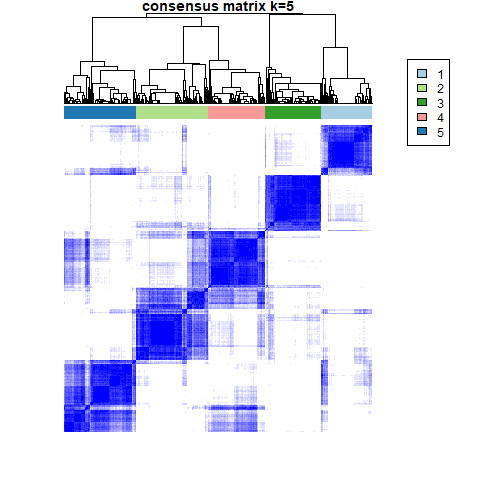

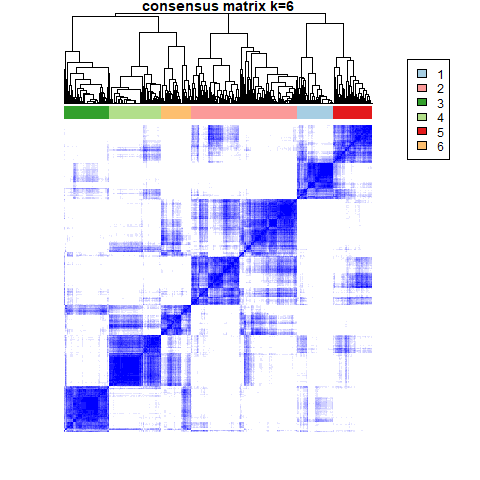

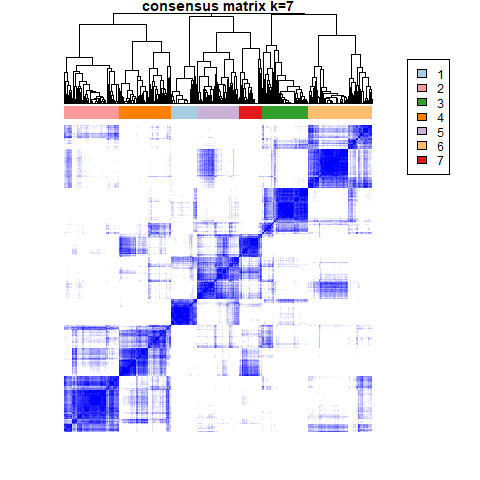

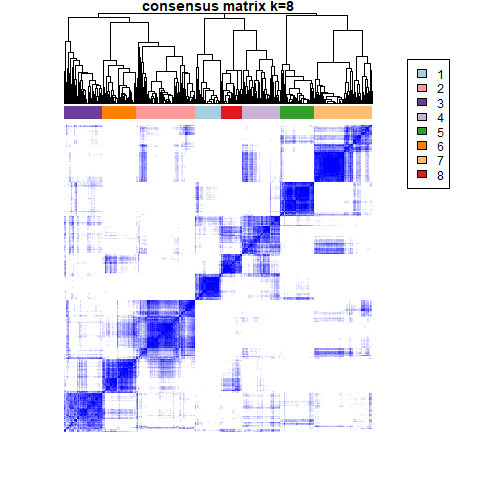

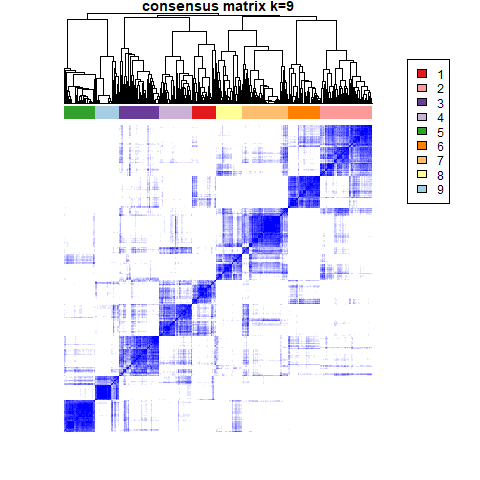


**Figure S4**


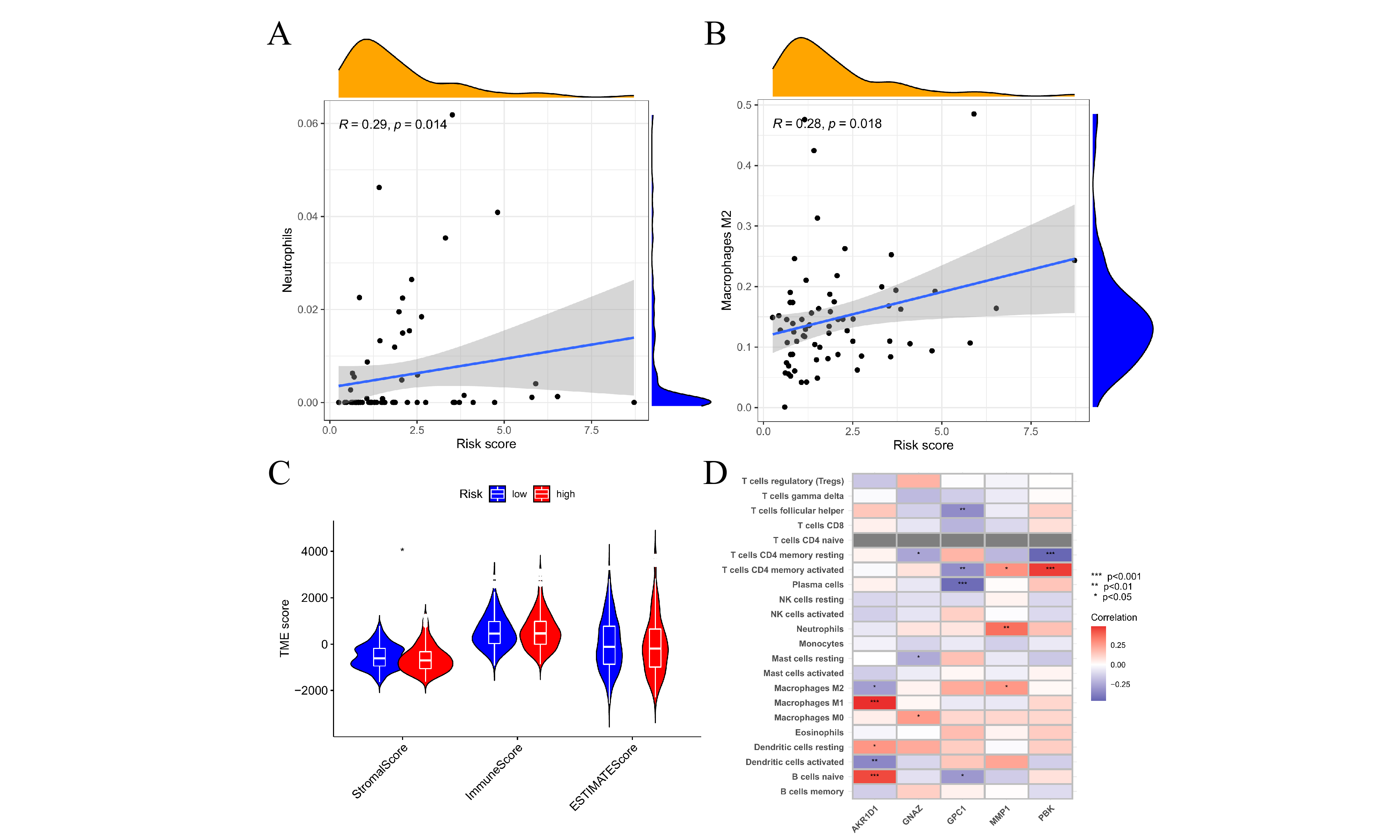


**Figure S5**


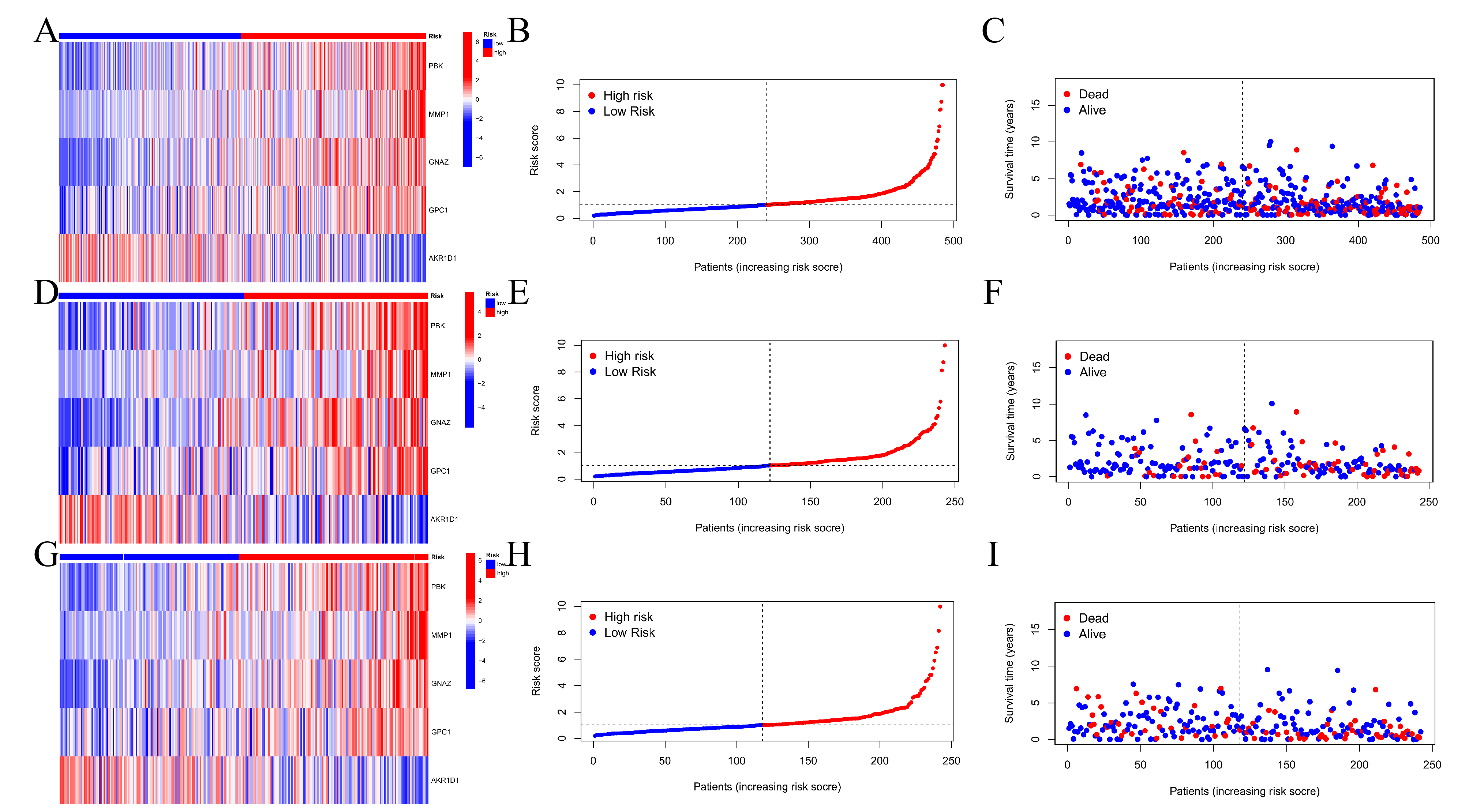

Supplement: Supplementary file 1 [file DataSheet_1.docx]
